# Reinforcement learning modulates the stability of cognitive control settings for object selection

**DOI:** 10.3389/fnint.2013.00095

**Published:** 2013-12-18

**Authors:** Anthony W. Sali, Brian A. Anderson, Steven Yantis

**Affiliations:** Department of Psychological and Brain Sciences, Johns Hopkins UniversityBaltimore MD, USA

**Keywords:** cognitive flexibility, reinforcement learning, attentional selection, decision making, impulsivity

## Abstract

Cognitive flexibility reflects both a trait that reliably differs between individuals and a state that can fluctuate moment-to-moment. Whether individuals can undergo persistent changes in cognitive flexibility as a result of reward learning is less understood. Here, we investigated whether reinforcing a periodic shift in an object selection strategy can make an individual more prone to switch strategies in a subsequent unrelated task. Participants completed two different choice tasks in which they selected one of four objects in an attempt to obtain a hidden reward on each trial. During a training phase, objects were defined by color. Participants received either consistent reward contingencies in which one color was more often rewarded, or contingencies in which the color that was more often rewarded changed periodically and without warning. Following the training phase, all participants completed a test phase in which reward contingencies were defined by spatial location and the location that was more often rewarded remained constant across the entire task. Those participants who received inconsistent contingencies during training continued to make more variable selections during the test phase in comparison to those who received the consistent training. Furthermore, a difference in the likelihood to switch selections on a trial-by-trial basis emerged between training groups: participants who received consistent contingencies during training were less likely to switch object selections following an unrewarded trial and more likely to repeat a selection following reward. Our findings provide evidence that the extent to which priority shifting is reinforced modulates the stability of cognitive control settings in a persistent manner, such that individuals become generally more or less prone to shifting priorities in the future.

## Introduction

An important component of adaptive behavior is the ability to flexibly update cognitive operations such as the deployment of attention or the selection of a behavioral strategy. Attentional selection is governed by cognitive control settings (e.g., Wolfe et al., 1989; Folk et al., 1992; Corbetta and Shulman, 2002) and determines which information from the environment receives cognitive processing and influences decision making (Yantis and Johnston, 1990; Desimone and Duncan, 1995; Reynolds et al., 1999; Yantis and Egeth, 1999). In order to promote survival and well-being, cognitive control settings must prioritize stimuli that will yield rewarding outcomes when selected. As task demands and reward contingencies change, individuals must be able to flexibly update these control settings. Converging evidence suggests that previous experiences as well as trait individual differences contribute to between-subject variance in cognitive flexibility (Hertwig et al., 2004; Cools, 2008; Hertwig and Ervev, 2009). In particular, search history influences future control settings both within the same task as well as across seemingly diverse domains of cognition (Hills and Hertwig, 2010; Hills et al., 2010). However, the degree to which the stability of an environment's reward structure may persistently influence future cognitive control states remains poorly understood. In the current study, we therefore examined whether the rate at which reward contingencies unpredictably changed in the past influences future selection behavior, making an individual more or less prone to switch strategies.

When searching for a hidden reward, individuals may choose to explore the environment by testing new behavioral selections or to exploit selections that were rewarded in the past (see Cohen et al., 2007). With limited time and resources, individuals must set a criterion for the amount of evidence required to stop selecting one option and begin selecting another in order to maximize reward. Recent evidence suggests that human observers follow Charnov's Marginal Value Theorem, a model of animal behavior, when searching for hidden rewards such that selection switches occur when the reward yield from the currently exploited selection falls below the overall average reward yield (Charnov, 1976; Wolfe, 2013). Individuals therefore tend to switch to an exploration strategy once an exploited selection begins to yield rewards at a rate that is below the expected value of the other options as a whole.

An individual's adoption of either exploration or exploitation strategies in the past primes future behavior. Hills and Hertwig (2010) had participants make selections between two alternatives with differing reward distributions. Participants were given feedback after each selection to allow for learning, but critically there were no monetary consequences based on participants' choices. Participants who frequently switched targets during this evaluation period tended to base a final consequential selection on discrete comparisons of individual trials and to underweight rare events. Conversely, those participants who did not switch frequently were more likely to choose whichever target had the overall larger average yield across the entire evaluation phase. Similarly, in another study, participants who engaged in exploitative search demonstrated more stable behaviors in a later lexical decision task than those who had previously engaged in explorative search (Hills et al., 2010). Taken together, both studies provide evidence that individuals' previous selection strategies modulate current states of cognitive flexibility.

Existing tasks such as those used by Hills et al. (2010) and Hills and Hertwig (2010) do not account for situations in which reward contingencies in the environment change periodically and without warning. Under such changing conditions, individuals must decide when to update their predictions regarding the value of each potential selection. In a dynamic environment, any non-rewarded selection may be indicative of a decrement in the true underlying value of the selected object to below that of alternatives, or could simply result from a probabilistic instance of no reward following optimal selection. A stable strategy of cognitive control in which a particular object remains prioritized despite periodically missed rewards may be advantageous when the object-reward contingencies remain reliable and consistent. At the same time, this stable strategy may be disadvantageous under conditions in which such contingencies are subject to change unpredictably. Consequently, in the current experiment, we exposed participants to either an initial learning environment in which reward contingencies were held constant, or an environment in which contingencies could change without warning.

Although the influence of previously experienced reward contingency stability on future states of cognitive control remains unclear, both attentional priority and cognitive control processes are sensitive to reward learning (e.g., Anderson et al., 2011b). Reward plays an important role in modulating attentional processing (e.g., Della Libera and Chelazzi, 2009; Raymond and O'Brien, 2009; Hickey et al., 2010a,b) and through associative learning can create persistent changes in the attentional priority of stimuli (Anderson et al., 2011a,b, 2012, 2013; Anderson and Yantis, 2012, 2013; see Anderson, 2013, for a review). Furthermore, Jimura et al. (2010) found that reward influences proactive cognitive control: participants made faster judgments in a working memory task for experimental blocks in which accurate performance was sometimes rewarded than in blocks for which there was no available reward. This reward-based facilitation in response time as well as a corresponding neural correlate of sustained proactive control were both positively associated with individual differences in reward sensitivity. In the current experiment, we extend these previous findings regarding the role of reward learning on attentional priority and cognitive control to examine whether persistent changes in the stability of cognitive control settings can result from learned expectations concerning the consistency of object-reward contingencies.

Individual differences serve as a second potential source of variability in cognitive selection strategies. In particular, the construct of impulsivity has been linked to variation in cognitive flexibility (e.g., Cools, 2008). Between-subject variation in impulsivity is attributed to concentrations of dopamine within the prefrontal cortex and striatum, which is governed by polymorphisms of the catechol-*O*-methyltransferase (COMT) and dopamine transporter (DAT) genes, respectively (Nolan et al., 2004; Bertolino et al., 2006; Cools, 2008; Bédard et al., 2010; Heatherton and Wagner, 2011). Although previous research has associated impulsivity with a range of behavioral deficits and disorders such as drug abuse (Hester and Garavan, 2004; Nielsen et al., 2012; Papachristou et al., 2012) and attention deficit hyperactivity disorder (ADHD; Cools, 2008; Bédard et al., 2010), healthy adults also demonstrate considerable variability in trait impulsivity (Patton et al., 1995). Individual differences in impulsivity are associated with a preference for immediate reward and may therefore influence individuals' willingness to switch behavioral strategies following an unrewarded selection (Barkley, 1997; Sonuga-Barke, 2003; Tripp and Wickens, 2008).

Given the existing evidence that previous experiences and trait impulsivity both influence future states of cognitive flexibility, in the current study, we investigated the unique contribution of both factors when accounting for behavioral selection strategies. Unlike previous studies of the impact of selection history on future control settings, we chose to manipulate across subjects the frequency with which participants needed to update reward predictions in an initial training phase. Specifically, we manipulated the frequency with which the more-highly rewarded object switched identity across participants. Participants selected a square on each trial, after which the location of a hidden reward was revealed. If they had selected the rewarded square, they obtained the reward. Half of the participants learned that the selection of a particularly colored square would lead to a monetary reward for the majority of trials throughout the entirety of the training phase. For the remainder of the participants, the most frequently rewarded color switched periodically and without warning. We refer to these two training conditions as stable and flexible, respectively. Immediately following the training phase, all participants completed a novel decision making task (test phase) in which the more-often rewarded object was defined by its spatial location. Critically, there was a consistent relationship between stimulus location and the likelihood of receiving reward for all participants, thus allowing comparison of choice strategy stability as a function of training history. First, we hypothesized that more impulsive individuals would make more variable choices during the test phase, being more influenced by recently missed rewards, regardless of training condition. Furthermore, we predicted that when statistically controlling for any variance in choice behavior associated with trait impulsivity, test phase selections would vary as a function of training history. Specifically, we predicted that participants in the stable training condition would engage in less variable choice behavior during the test phase and be less likely to switch object selections on a trial-by-trial basis than those in the flexible training group, reflecting a persistent shift in cognitive flexibility.

## Materials and methods

### Participants

Sixty-two individuals (41 females) ranging in age from 18 to 33 (*M* = 21.3, *SD* = 3.21) completed the study in exchange for monetary compensation. All participants signed a consent form that was approved by the Johns Hopkins University Institutional Review Board. Participants were randomly assigned to the stable and flexible training groups. Data from one participant was excluded due to prior participation in a pilot study involving the same test phase. Data from a second participant was also excluded from all analyses because they produced no variability in selection (the same object was selected on every trial) in both the training and test phases despite being in the flexible training group.

### Apparatus

Participants were seated facing an Asus VE247 LCD monitor that was connected to a Mac Mini computer. Stimulus presentation and response collection was controlled by the Psychophysics toolbox for Matlab (Brainard, 1997). The monitor was positioned approximately 76 cm from the participant. Participants made all responses during the training phase using a standard computer mouse. Responses during the test phase were made using the four arrow keys of a standard keyboard. Both the keyboard and the mouse were positioned on a table in front of the participant.

### Stimuli

#### Training phase

**Figure 1:**
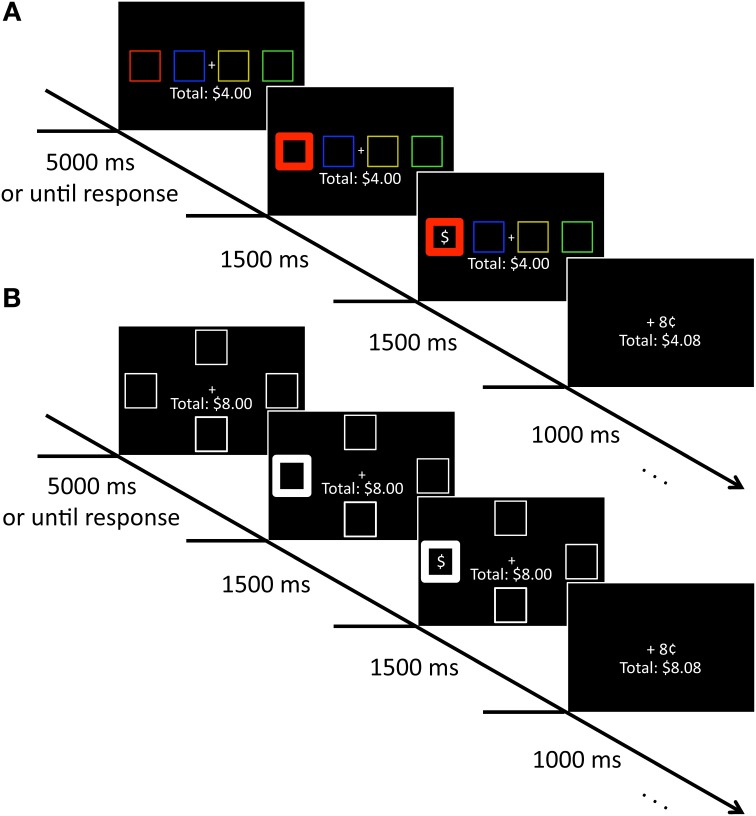
**Sequence of events for training and test phase trials. (A)** During training, participants selected a colored square to uncover a hidden reward. Participants accumulated money for each reward-containing square they selected. **(B)** During test, participants selected a square based on location. As in the training phase, they accumulated money for selecting a square containing a hidden reward.

On each trial, four colored squares (each 2.03 × 2.03^°^ visual angle with an 2.03° gap between stimuli edge-to-edge) appeared along the horizontal meridian of the computer screen against a black background. These squares were positioned to the left and right of a central crosshairs and were rendered in red, green, blue, and yellow. A running total of the participant's earnings in the study was continuously displayed beneath the crosshairs, centered on the vertical meridian (see Figure 1A). After the selection of a square, reward feedback followed that consisted of a dollar sign appearing in one of the four squares along with either “+8¢” or “+0¢” indicated above the total earnings. The selected square became bold (line width increased from 1 to 10 pixels) to indicate its selection.

#### Test phase

The stimuli were identical to those in the training phase with the exception that all four squares were white and positioned above, below, to the left, and to the right of the central crosshairs (7.17° center-to-center). A running total of the participant's earnings was again continuously displayed beneath the crosshairs, and the same feedback sequence again followed selection of a square (see Figure 1B).

#### Barratt impulsivity scale

Participants completed the Barratt Impulsivity Scale (BIS-11; Patton et al., 1995). All but two participants completed the measure immediately prior to the training phase of the experimental task; one completed the BIS-11 5 days prior to participating in the current experiment and the other completed it 2 days after participating in the experimental task. The BIS-11 consists of 30 items such as “I say things without thinking,” and “I act on the spur of the moment.” For each item, participants rated the degree to which they engaged in the described behavior on a four-point scale ranging from (1) “Rarely/Never” to (4) “Almost Always.” We computed the total impulsivity score for each participant by summing the responses to all items. Omitted items on the questionnaire were assigned that subject's mean response; items for which a single participant selected more than one response were assigned the average of the two items. Scores on the BIS-11 ranged from 46 to 88 (*M* = 59.42, *SD* = 9.02).

### Procedure

#### Training phase

Each of 240 trials began with the presentation of four colored squares, the arrangement of which was randomly determined. Participants moved a cursor on the screen using a computer mouse and selected a single square by clicking the left mouse button. Clicks outside of a colored square were not counted or recorded. Following the mouse click, the selected square's color outline was bolded for 1.5 s to indicate to the participant that the selection was registered. Next, a dollar sign appeared inside one of the four squares and was presented along with the bold outline of the selected square for an additional 1.5 s. If the participant had selected the square that had the dollar sign, 8¢ was added to their total earnings. Participants viewed reward feedback of either “+8¢” or “+0¢” for 1 s following the presentation of the dollar sign (see Figure 1A). At no point during the task was fixation enforced. If participants did not make a selection within 5 s, all of the squares became bolded for 1.5 s. All other aspects of the trial were the same as in those in which a response was made.

We manipulated, between subjects, the likelihood that each square would receive the hidden reward. For half of the participants (the stable training group), selection of a single color (counterbalanced across participants) was associated with the receipt of the reward on 70% of the training phase trials. The remaining three colored squares each contained the hidden reward on 10% of all trials. Participants in this first condition therefore received consistent contingencies in which one color was always the most likely to contain the hidden reward. Conversely, the remainder of participants received a flexible training schedule in which the color frequently containing the hidden reward was updated periodically and without notification. For participants in this condition, each of the four colored squares contained the hidden reward for 70% of all trials occurring during one of four 60 trial blocks. There was no break between blocks to indicate to participants when this switch occurred and we counterbalanced which color was most-often rewarded in the first block. The order in which the remaining colors were most-often rewarded was consistent across participants such that the order was always red, yellow, blue, green (red followed green such that one potential order was blue, green, red, yellow).

#### Test phase

Immediately following the training phase, all participants completed a 240 trial test phase in which we examined whether training history influenced choice behavior. Participants selected squares based on location using the four arrow keys of a standard keyboard (e.g., the right arrow key selected the square to the right of the central crosshairs). As in the training phase, the outline of the selected square became bold for 1.5 s and then a dollar sign was presented inside one of the squares for 1.5 s (see Figure 1B). Reward feedback was presented for 1 s prior to the onset of the next trial. All of the squares became bolded for 1.5 s following trials in which participants failed to make a selection within 5 s. A single square location (counterbalanced across participants) contained the hidden reward on 40% of all test phase trials; the remaining three locations each contained the hidden reward on 20% of trials. We set the reward contingencies among the four squares to be more similar in the test phase than in the training phase to make the optimal strategy less clear for the participants. Following completion of the test phase, participants were debriefed.

## Results

### Training phase

**Figure 2:**
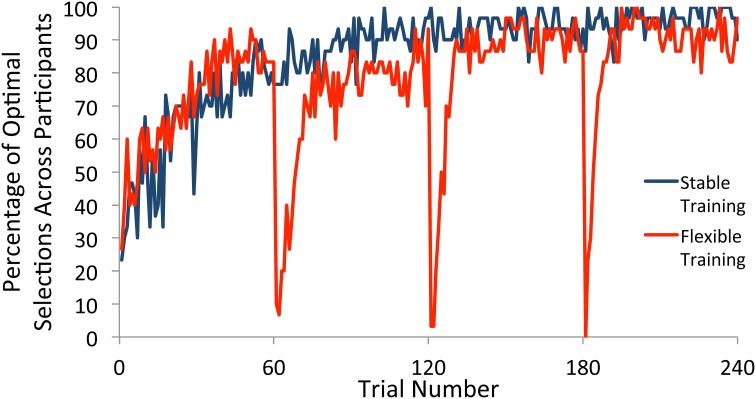
**Percentage of participants who selected the optimal square on each trial of the training phase**. Participants who received flexible training quickly adjusted their behavior to the new reward contingencies within each block after a switch. Participants who received stable training maintained a single selection rule throughout the task.

We first examined choice behavior in the training phase. For participants in the flexible training group, there were three switches in the underlying reward structure, with each occurring after 60 trials of consistent reward contingencies. For each trial, we computed the percentage of participants who selected the most frequently rewarded square (referred to here as the optimal selection) within each block. The percentage of optimal selections across individuals on any trial therefore provided an estimation of when participants had converged on the optimal strategy according to the current contingencies. As illustrated in Figure 2, participants in both the flexible and stable conditions quickly learned the selection rule, settling on the optimal square. With the start of a new block, participants in the flexible training condition quickly adapted to the new reward contingencies and showed a strong tendency to select the newly-defined optimal square after a small number of trials. This rapid adjustment of behavior in response to a shift in reward contingencies provides evidence that our manipulation was effective.

### Test phase

**Figure 3:**
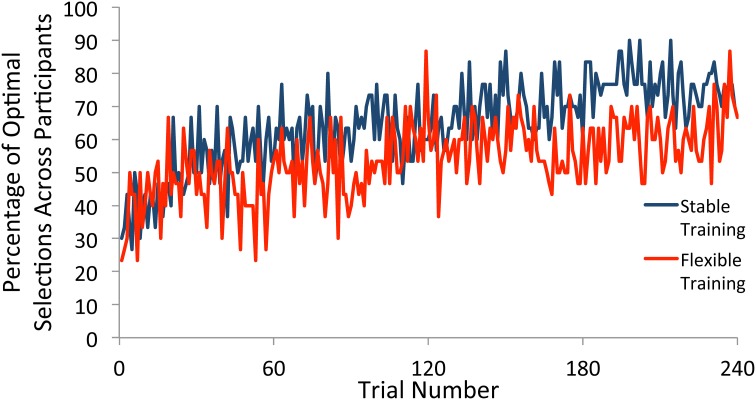
**Percentage of participants who selected the optimal square on each trial of the test phase**. Participants selected the optimal target at a greater rate than would be expected due to random guessing throughout the test phase following both stable and flexible training.

We next plotted the percentage of participants who selected the optimal target according to the test phase probabilities for each trial (see Figure 3). In order for any observed differences in how frequently participants deviate from the optimal strategy as a function of training condition to be meaningful, it is important that both training groups show evidence of rule learning. To determine the time course of rule learning in the test phase, we split the data into groups of 60 trials each. Beginning with the first 60 trials, and continuing throughout the entirety of the test phase, participants in both training groups were substantially more likely to pick the optimal target than would be expected if they had made all selections randomly (*p*'s < 0.001), demonstrating learning.

We computed three measures of choice flexibility to determine whether performance in the test phase varied as a function of the reward contingencies experienced during the training phase. First, we computed an index of choice variability for each participant, here referred to as the choice stability index. To compute the choice stability index, we calculated the total number of selections for each of the location-defined squares across the entire test phase. We then computed the standard deviation of the four square-selection sums as a measure of choice variability. A greater choice stability index means that a participant tended to select certain squares more frequently than others, while a low choice stability index reflects a more equal spread of selections across the four squares.

For the remaining two measures of choice flexibility, we categorized trials based on whether the participant's selection on the previous trial was rewarded. Out of the total number of trials following an unrewarded selection, we computed the percentage for which participants selected an object that differed from their previous selection. Similarly, for trials following a rewarded selection, we calculated the percentage of trials for which participants selected the same object as they had on the previous trial.

**Figure 4:**
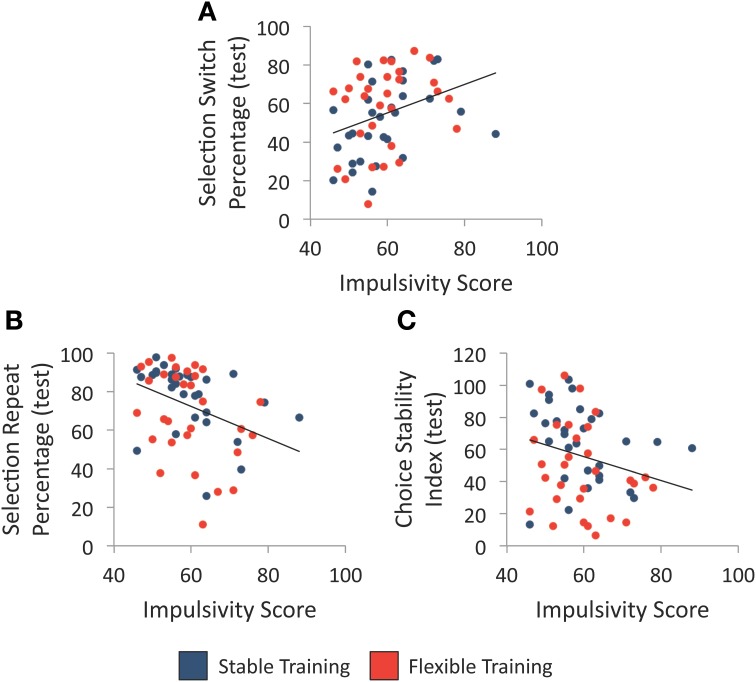
**Individual differences in trait impulsivity and selection behavior. (A)** Relationship between individual differences in trait impulsivity and the percentage of object selection switches following an unrewarded selection during test. **(B)** Relationship between individual differences in trait impulsivity and the percentage of object selection repeats following a rewarded selection during test. **(C)** Relationship between individual differences in trait impulsivity and choice stability index during test. Each line denotes the best-fit linear regression equation when collapsing across training groups.

We first examined whether individual differences in impulsivity, as assessed with the BIS-11, were associated with variability in each of the three choice flexibility measures regardless of training history. As illustrated in Figures 4A,B, high impulsivity was associated with a greater percentage of selection switches following unrewarded selections, *r*_(58)_ = 0.32, *p* = 0.012, as well as a smaller percentage of selection repeats following rewarded selections, *r*_(58)_ = −0.36, *p* = 0.005. Furthermore, there was a trend between trait impulsivity and choice stability index scores, *r*_(58)_ = −0.25, *p* = 0.055, such that participants with greater trait impulsivity tended to make less stable selections during the test phase (see Figure 4C). Given the relationship between impulsivity and choice flexibility regardless of training condition, we report all group comparisons below with impulsivity score entered as a covariate to determine whether group differences exist when statistically controlling for individual differences in impulsivity.

**Figure 5:**
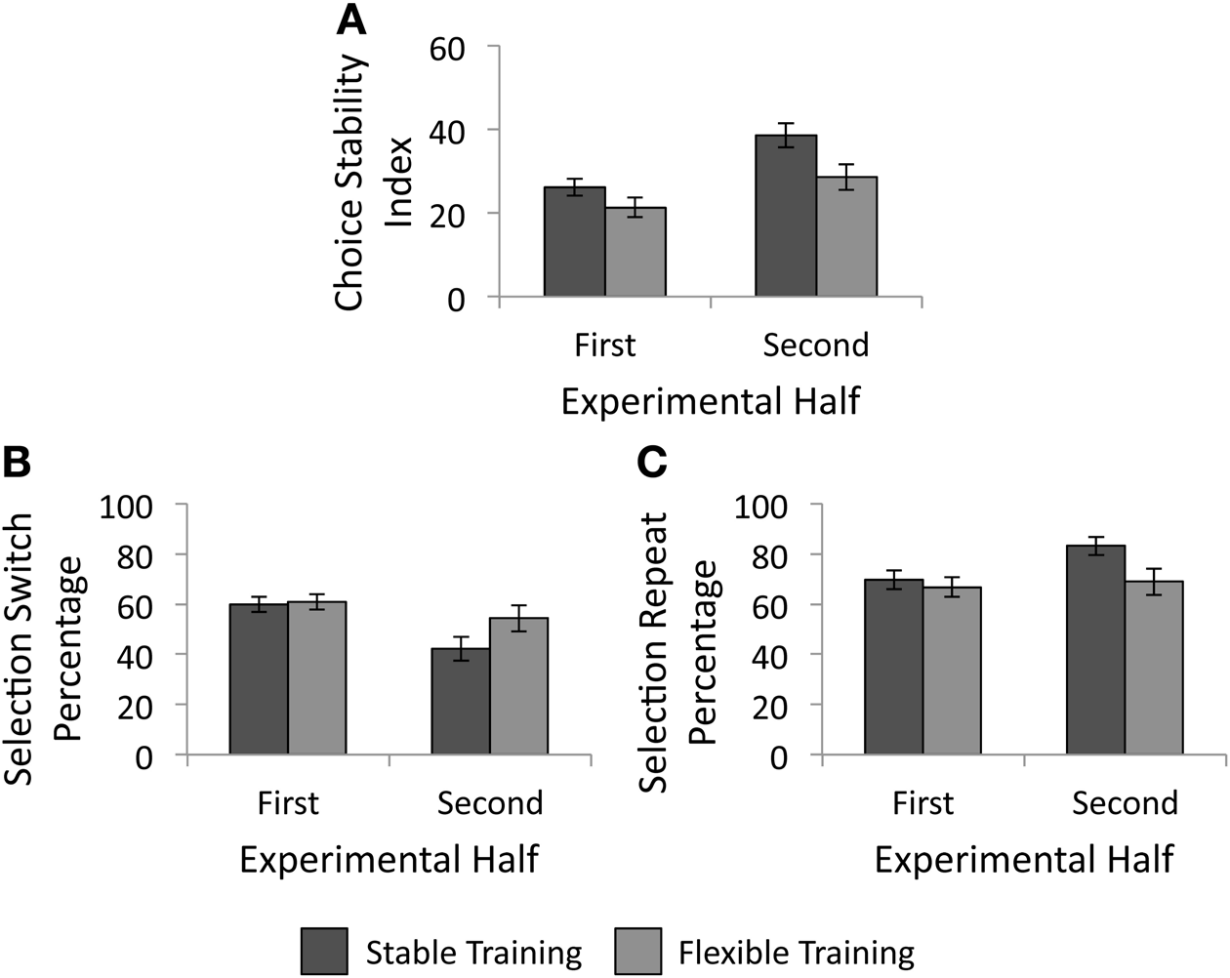
**Behavioral results from the test phase. (A)** Choice stability index as a function of training group. Participants continued to make less variable responses following the stable training than following the flexible training. **(B)** Percentage of trials following an unrewarded selection in which participants selected a different object than their previous selection, as a function of training group. **(C)** Percentage of trials following a rewarded selection in which participants made the same selection as they had on the previous trial, as a function of training group. Error bars denote 1 SE above and below the mean.

We conducted a 2 × 2 analysis of covariance (ANCOVA) with factors of training condition (stable vs. flexible) and experimental half (first vs. second) to determine the impact of reward history on future selection strategies. As mentioned above, impulsivity scores were entered into the model as a covariate. As illustrated in Figure 5A, there was a significant main effect of training condition, *F*_(1, 57)_ = 5.38, *p* = 0.024, such that participants made more variable selections in the test phase following the flexible training than following stable training. The main effect of experimental half failed to reach significance, *F*_(1, 57)_ < 0.01, *p* = 0.957, as did the interaction of experimental half and training condition, *F*_(1, 57)_ = 2.21, *p* = 0.142. As hypothesized, individuals who received unpredictably changing reward contingencies in the past made more variable selections during the test phase object selection task.

We next examined whether training history influenced the likelihood that individuals selected a different square than they had on the previous trial. First we investigated whether the percentage of selection switches made following an unrewarded selection varied as a function of training history or experimental half with a 2 × 2 ANCOVA with impulsivity added as a covariate. There were no significant main effects of training condition, *F*_(1, 57)_ = 1.69, *p* = 0.199, or experimental half, *F*_(1, 57)_ = 0.70, *p* = 0.408. As illustrated in Figure 5B, there was a significant interaction between experimental half and training condition, *F*_(1, 57)_ = 5.53, *p* = 0.022, such that although there was little difference between groups in the likelihood to switch following an unrewarded selection in the first half, a difference emerged in the second half. Participants who received stable training were less likely to switch following an unrewarded selection in the second half than participants who received flexible training. Next, we tested whether the percentage of selection repeats following a rewarded selection varied as a function of training history or experimental half with another 2 × 2 repeated measures ANCOVA. As above, impulsivity scores were again entered into the model as a covariate. The main effect of training condition, *F*_(1, 57)_ = 3.12, *p* = 0.083, as well as the main effect of experimental half, *F*_(1, 57)_ = 0.27, *p* = 0.605, again failed to reach statistical significance. However, there was a significant interaction between training condition and experimental half, *F*_(1, 57)_ = 4.55, *p* = 0.037, such that a group difference again emerged in the second half of the test phase (see Figure 5C). Participants who received stable training were more likely to repeat a selection following a rewarded trial than those who received flexible training.

**Figure 6:**
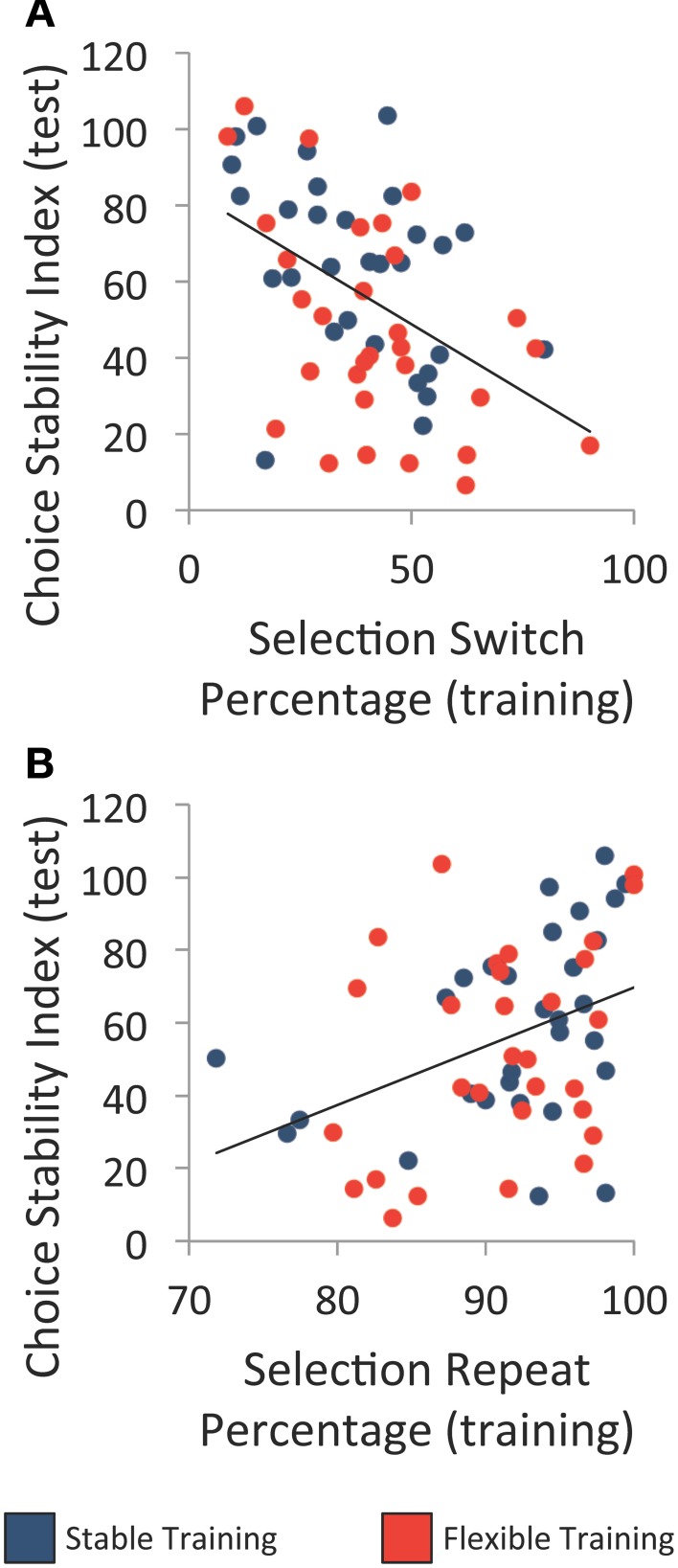
**Comparison of individuals' performance during training and test. (A)** Relationship between the percentage of object selection switches following an unrewarded selection during training and choice stability index at test. **(B)** Relationship between the percentage of object selection repeats following a rewarded selection during training and choice stability index at test. Each line denotes the best-fit linear regression equation when collapsing across training groups.

Lastly, we examined the relationship between switching behavior in the training phase and choice variability in the test phase collapsed across both halves. There was a negative correlation between the percentage of switches made following unrewarded selections during training and the choice stability index during test, *r*_(58)_ = −0.48, *p* < 0.001, and a positive correlation between the percentage of selection repeats following rewarded selections during training and the choice stability index during test, *r*_(58)_ = 0.38, *p* = 0.003 (see Figures 6A,B). These relationships were not specific to either the flexible or stable training condition, as indicated by direct comparison (*p*'s > 0.780). These correlations indicate that on an individual level, shifting strategy more often during training was associated with more variable selections during test.

## Discussion

In the current study, we examined whether the consistency of previous reward contingencies in a choice task influenced later selection strategies independent of individual differences in impulsivity. Across two phases of the experiment, participants selected stimuli in an attempt to acquire a hidden reward. We found that individual differences in trait impulsivity accounted for variability in selection behavior, such that participants with high trait impulsivity were more likely to switch selections following a trial in which they did not receive a reward and less likely to select the same object again after receiving reward than participants with low impulsivity. Furthermore, although the reward contingencies were identical for all participants in the test phase of the experiment, selection patterns differed as a function of reward history. When statistically controlling for impulsivity, we found that participants who learned that the reward contingencies changed periodically and without warning in the training phase (flexible training group) made more variable selections in the test phase than those who experienced consistent training contingencies (stable training group). Furthermore, we found evidence in the second half of the test phase that flexible training participants were more likely to switch object selections on a trial-by-trial basis regardless of whether their selection was rewarded on the previous trial.

Our results suggest that the consistency of reward contingencies in the past influenced the weighting of reward outcomes for guiding behavior in the test phase. Participants did not vary in the likelihood that they switched object selections on a trial-by-trial basis when collapsing across the entire test phase. However, we found an interaction with experimental half for both measures such that participants who received the consistent reward contingency training demonstrated a greater shift toward stable behavior as the experiment progressed than participants who received the changing reward consistency training. Performance was similar early in the test phase, as participants gained experience with the current location-based reward contingencies. As the test phase progressed and learning continued, participants could develop expectations concerning the underlying reward structure to guide behavior. Our results suggest that the gradual accumulation of evidence that a consistent object was more-often rewarded in the test phase was weighted differently in the determination of strategy selection depending on training history. Participants who received stable training were more likely to adopt and maintain a stable test phase selection strategy in response to the consistent contingencies despite periodically missed rewards.

Our findings are consistent with recent research on search and decision making in humans (Hertwig et al., 2004; Hertwig and Ervev, 2009; Hertwig and Pleskac, 2010; Hills and Hertwig, 2010; Hills et al., 2010). Consistent with Hills and Hertwig (2010), we found that the experience of switching during an initial learning phase influenced later decisions. A difference between our paradigm and the search task used by Hills et al. (2010) was the content of the reward learning. Although participants learned to either exploit a single area or explore a wider range of areas in Hills and colleagues' previous search task, participants in the current study learned the likelihood that they would need to update a behavioral selection strategy. Monetary reinforcement therefore facilitated learning regarding the stability of the environment. Collectively, these findings provide converging evidence in favor of domain general reward-based modulations of cognitive control.

Our findings also build on recent research tying reward learning to the control of attention (e.g., Anderson et al., 2011b). The voluntary and involuntary selection of objects based on reward history has been a topic of considerable interest in investigations of both animal and human cognition (Glimcher, 2003; Della Libera and Chelazzi, 2009; Peck et al., 2009; Raymond and O'Brien, 2009; Gottlieb and Balan, 2010; Hickey et al., 2010a,b; Anderson et al., 2011a,b, 2012, 2013; Louie et al., 2011; Anderson and Yantis, 2012, 2013). The results of the current study provide evidence that reward history not only serves a modulatory role for computations of attentional priority, but also modulates the flexibility of cognitive control. Importantly, in the current study, participants did not learn to associate value with any particular stimulus feature. Rather, participants across the two training groups learned the consistency of reward contingencies. This learned knowledge from the training phase influenced object selection in a novel test phase. Our findings therefore suggest that monetary reinforcement may modulate attentional selection and decision making even when reward learning is not directly tied to a stimulus feature.

Reward-based modulations of cognitive flexibility have important implications for the study of top-down attentional control. Sustained and transient components of cognitive control are sensitive to task demands as well as reward-induced motivation (Botvinick et al., 2001; Braver et al., 2003; Brown and Braver, 2005; Jimura et al., 2010). Furthermore, control processes are known to fluctuate such that individuals are at times in a greater state of preparation to perform a cognitive operation such as a task switch or shift of spatial attention (Leber et al., 2008; Leber, 2010). The results of our study suggest that reward history also influences preparatory cognitive control. Thus, reward learning may serve as one additional mechanism through which individuals update preparatory control based on previous experiences.

The results of the current study also have important implications for understanding deficits of attentional control, such as ADHD and drug abuse, in which individuals demonstrate a sensitivity to immediate rather than delayed reward (Barkley, 1997; Cools, 2008). Given our findings that reward history influences the flexibility of goal-directed selection, such sensitivity to reward may contribute to large modulations of cognitive control based on previous experiences. Furthermore, we found evidence that trait impulsivity scores predicted participants' tendency to switch selections, and deficits in impulsiveness have been linked to both ADHD (e.g., Barkley, 1997; Mostofsky and Simmonds, 2008) and drug addiction (e.g., Hester and Garavan, 2004; Nielsen et al., 2012; Papachristou et al., 2012). Future research is needed to explore how dopaminergic dysfunctions in disorders such as ADHD (e.g., Bédard et al., 2010; Heatherton and Wagner, 2011), drug abuse (e.g., Volkow et al., 2009), and obesity (e.g., Volkow et al., 2011) are related to individual differences in impulsivity in healthy individuals and whether the neural mechanisms implicated in these disorders are influenced by rewards linked with states of cognitive control.

### Conflict of interest statement

The authors declare that the research was conducted in the absence of any commercial or financial relationships that could be construed as a potential conflict of interest.
